# Prediction of DNA-binding residues from protein sequence information using random forests

**DOI:** 10.1186/1471-2164-10-S1-S1

**Published:** 2009-07-07

**Authors:** Liangjiang Wang, Mary Qu Yang, Jack Y Yang

**Affiliations:** 1 Department of Genetics and Biochemistry, Clemson University, Clemson, SC 29634, USA; 2 J.C. Self Research Institute of Human Genetics, Greenwood Genetic Center, Greenwood, SC 29646, USA; 3 National Human Genome Research Institute, National Institutes of Health (NIH), U.S. Department of Health and Human Services, Bethesda, MD 20852, USA; 4 Harvard Medical School, Harvard University, P.O. Box 400888, Cambridge, MA 02115, USA

## Abstract

**Background:**

Protein-DNA interactions are involved in many biological processes essential for cellular function. To understand the molecular mechanism of protein-DNA recognition, it is necessary to identify the DNA-binding residues in DNA-binding proteins. However, structural data are available for only a few hundreds of protein-DNA complexes. With the rapid accumulation of sequence data, it becomes an important but challenging task to accurately predict DNA-binding residues directly from amino acid sequence data.

**Results:**

A new machine learning approach has been developed in this study for predicting DNA-binding residues from amino acid sequence data. The approach used both the labelled data instances collected from the available structures of protein-DNA complexes and the abundant unlabeled data found in protein sequence databases. The evolutionary information contained in the unlabeled sequence data was represented as position-specific scoring matrices (PSSMs) and several new descriptors. The sequence-derived features were then used to train random forests (RFs), which could handle a large number of input variables and avoid model overfitting. The use of evolutionary information was found to significantly improve classifier performance. The RF classifier was further evaluated using a separate test dataset, and the predicted DNA-binding residues were examined in the context of three-dimensional structures.

**Conclusion:**

The results suggest that the RF-based approach gives rise to more accurate prediction of DNA-binding residues than previous studies. A new web server called BindN-RF  has thus been developed to make the RF classifier accessible to the biological research community.

## Background

Many nuclear proteins perform essential functions through interaction with DNA. For instance, transcription factors activate or repress downstream gene expression by binding to specific DNA motifs in promoters [1]. To understand the molecular mechanism of protein-DNA recognition, it is important to identify the DNA-binding residues in proteins. The identification is straightforward if the structure of a protein-DNA complex is known. However, it is rather expensive and time-consuming to solve the structure of a protein-DNA complex. Currently, only a few hundreds of protein-DNA complexes have structural data available in the Protein Data Bank [2]. With the rapid accumulation of sequence data from many genomes, computational methods are needed for accurate prediction of DNA-binding residues in protein sequences. The prediction results can provide useful information for protein functional annotation, protein-DNA docking, and experimental studies such as site-directed mutagenesis.

Machine learning is particularly appealing for modelling the DNA-binding pattern of amino acid residues. Although some experimental observations have been made for DNA-binding residues in protein structures, the molecular recognition pattern is still poorly understood [3]. It is thus desired that machine learning methods can be used to model the complex patterns hidden in the available structural data, and the resulting classifier can be applied to reliable identification of DNA-binding residues in protein sequences. The machine learning problem can be formally specified as follows: given the amino acid sequence of a protein that is supposed to interact with DNA, the task is to predict which amino acid residues may be located at the interaction interface. Since both the structure of the protein and the sequence of the target DNA are assumed to be unknown, it is challenging to predict DNA-binding residues from amino acid properties and local sequence patterns.

Several machine learning methods have been reported for predicting DNA-binding residues in protein sequences. Ahmad et al. [4] analyzed the structural data of representative protein-DNA complexes, and used the amino acid sequences in these structures to train artificial neural networks (ANNs) for DNA-binding site prediction. Yan et al. [5] constructed Naïve Bayes classifiers using the amino acid identities of DNA-binding sites and their sequence neighbours. However, the prediction accuracy was relatively low in these studies [4,5], probably because amino acid sequences were directly used for classifier construction.

The use of domain-specific knowledge for input encoding has been shown to enhance classifier performance. Ahmad and Sarai [6] developed an ANN-based method to utilize evolutionary information in terms of position-specific scoring matrices (PSSMs). The scores in a PSSM indicate how well each amino acid position of a sequence is conserved among its homologues. Since functional sites tend to be conserved among homologous proteins, PSSMs may provide relevant information for predicting DNA-binding residues. It was found that the average of sensitivity and specificity could be increased by up to 8.7% using PSSMs when compared with ANN predictors using sequence information only [6]. More recently, PSSMs were also used to train support vector machines (SVMs) and logistic regression models for accurate prediction of DNA-binding residues [7,8].

In our previous studies [9,10], ANN and SVM classifiers were constructed using relevant biochemical features, including the hydrophobicity index, side chain pK_a _value, and molecular mass of an amino acid. These features were used to represent biological knowledge, which might not be learned from the training data of DNA-binding residues. It was found that classifier performance was significantly improved by the use of biochemical features for input encoding, and the SVM classifier outperformed the ANN predictor [9,10].

There are two main objectives of the present study. The first objective is to improve the prediction accuracy by combining different types of biological knowledge in classifier construction. Although either PSSMs or biochemical features have been used for input encoding, it is still unknown whether classifier performance can be further improved through a combination of relevant features, including new descriptors of evolutionary information. One potential problem is that the use of PSSMs for classifier construction gives rise to a large number of input variables. A training data instance normally includes multiple neighbouring residues for providing context information, and each residue has 20 PSSM scores. Considering the relatively small dataset currently available for modelling DNA-binding sites, too many input variables may result in model overfitting for most machine learning algorithms. Thus, the second objective is to investigate whether accurate classifiers can be constructed using the random forest (RF) learning algorithm, which has the capability to handle a large number of input variables and avoid model overfitting [11]. The results obtained in this study indicate that DNA-binding site prediction can be significantly improved by using the RF-based approach with biochemical features and several new descriptors of evolutionary information for input encoding.

## Methods

### Data preparation

This study used two amino acid sequence datasets, PDNA-62 and PDC25t, which were extracted from the structural data of protein-DNA complexes available at the Protein Data Bank . The PDNA-62 dataset was used classifier construction in this work as well as several previous studies [6-10]. The amino acid sequences in PDNA-62 were derived from 62 structures of representative protein-DNA complexes, and the dataset had less than 25% identity among the sequences. The PDC25t dataset was derived from the protein-DNA complexes that were not included in PDNA-62. The sequences in PDC25t had less than 25% identity among them as well as with the sequences in PDNA-62. In this study, PDC25t was used as a separate test dataset for classifier performance evaluation and comparison.

As in our previous studies [9,10], an amino acid residue was designated as a binding site if the side chain or backbone atoms of the residue fell within a cutoff distance of 3.5 Å from any atoms of the DNA molecule in the complex. All the other residues were regarded as non-binding sites. Both PDNA-62 and PDC25t are imbalanced datasets with ~15% residues labelled as DNA-binding and ~85% residues being non-binding.

### Training strategies

Classifiers were trained using residue-wise data instances derived from the sequence dataset (PDNA-62). Each data instance had eleven consecutive residues, and the target residue was positioned in the middle of the subsequence. A data instance was labelled as positive if the target residue was DNA-binding, or negative if the target residue was non-binding. The context information provided by the five neighbouring residues on each side of the target residue was previously shown to be optimal for sequence-based prediction of DNA-binding residues [9,10].

In classifier construction, the input vector was generated by encoding each residue with three biochemical features and several descriptors of evolutionary information (see below). In our previous studies [9,10], the three biochemical features, including the hydrophobicity index (feature *H*), side chain pK_a _value (feature *K*), and molecular mass (feature *M*) of an amino acid, were shown to be relevant for DNA-binding site prediction.

### Evolutionary information extraction

For DNA-binding site prediction, the labelled datasets derived from the available structures are relatively small in size. However, there are abundant unlabeled sequence data in public databases such as UniProt [12]. The unlabeled data contain evolutionary information about the conservation of each sequence position. Because DNA-binding residues tend to be conserved among homologous proteins [13], evolutionary information can be used to enhance classifier performance.

**Figure 1:**
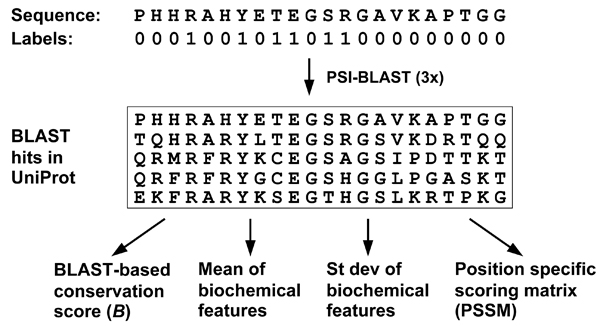
**Schematic diagram for extracting evolutionary information from the PSI-BLAST search result**.

The procedure for extracting evolutionary information from sequence alignments is outlined in Figure 1. For a given protein sequence *p*, its homologues in a reference database can be retrieved and aligned to *p *using the PSI-BLAST program [14]. The sequence alignment is then used to compute evolutionary conservation scores for each residue in *p*. In this study, the protein sequence dataset UniProtKB  was used as the reference database, and PSI-BLAST was run for three iterations with the E-value threshold set to 1e-5. The following descriptors of evolutionary information have been investigated for DNA-binding site prediction:

(1) BLAST-based conservation score (feature *B*): Let *H*_*p *_= {*h*_1_, *h*_2_,..., *h*_*n*_} be the set of *n *hits (*n *> 0) in the PSI-BLAST search for a query sequence *p*. Each hit is a pair-wise sequence alignment, in which PSI-BLAST indicates whether two aligned residues are identical or show similarity based on the BLOSUM62 scoring matrix [14]. The *B *score for the residue *a*_*i *_at position *i *in *p *is computed as follows:

(1)

where *f *(*a*_*i*_, *h*_*j*_) is set to 1 if *a*_*i *_is aligned to an identical or similar residue in *h*_*j*_, or 0 otherwise, and *c *is a pseudo-count (set to 10 in this work). The term (*c*/*n*) is used to scale the feature value, and it becomes smaller when *n *gets larger. If *p *has no hit in the database (*n *= 0), the feature value is set to 0. The *B *score was used to construct artificial neural network classifiers in our previous study [9].

(2) Mean and standard deviation of biochemical feature values: For each residue *a*_*i *_in the sequence *p*, the mean () and standard deviation (*σ*) of a biochemical feature *X*, *X *∈ {*H*, *K*, *M*}, are calculated as follows:

(2)

(3)

where *χ*(*a*_*i*_, *h*_*j*_) is the value of feature *X *for the amino acid residue in *h*_*j*_, which is aligned to *a*_*i *_at position *i *in *p*. The mean of feature *X*, also referred to as *H*_*m*_, *K*_*m *_or *M*_*m *_in this paper, captures the biochemical properties of an amino acid position in the sequence alignment. It has been shown that basic and polar amino acids are overrepresented while acidic and hydrophobic amino acids are underrepresented in the population of DNA-binding sites [4,9]. The standard deviation of feature *X*, also called *H*_*d*_, *K*_*d *_or *M*_*d*_, reveals how well the biochemical properties of an amino acid position are conserved in the aligned homologous sequences.

(3) Position-specific scoring matrix (PSSM): The PSSM scores are generated by PSI-BLAST [15], and there are 20 values for each sequence position. The evolutionary information captured by PSSMs was previously shown to improve the performance of artificial neural networks and support vector machines for DNA-binding site prediction [6,7]. However, PSSM is rather designed for BLAST searches, and it may not capture all the evolutionary information for modelling DNA-binding sites.

### Random forests

The use of evolutionary information for classifier construction results in a large number of input variables. In particular, since PSSM has 20 scores for each sequence position, it gives rise to 220 inputs for a data instance with eleven residues. Considering the relatively small size of the training dataset, too many inputs may result in model overfitting. In this study, we used the random forest learning algorithm, which was shown to have the capability of handling a large number of input variables and avoiding model overfitting [11].

Random forests (RFs) use a combination of independent decision trees to improve classifier performance. Specifically, each decision tree in a forest is constructed using a bootstrap sample from the training data. During tree construction, *m *variables out of all the *n *input variables (*m *<<*n*) are randomly selected at each node, and the tree node is split using the selected *m *variables. Because of the random feature selection, RFs can handle a large number of input variables and avoid overfitting. For classifying a data instance, a RF classifier combines the votes made by the decision trees, and gives the most popular class as the output of the ensemble. It has been shown that RFs outperform AdaBoost ensembles on noisy datasets, and can work well on data with many weak inputs [11]. These characteristics of RFs are appealing since the DNA-binding data appear to be noisy and contain many weak sequence-derived features.

In this study, we used the software package available at  construct RF classifiers with the default parameter settings. In particular, the number of variables selected to split each node (*m*) was set to the floor of square root of the total number of input variables. Other values of *m *were also tested, but did not result in significant improvement of classifier performance for DNA-binding site prediction.

### Classifier evaluation

We performed fivefold cross-validation experiments using the PDNA-62 dataset for the initial estimation of classifier performance. The trained classifier was further evaluated using the PDC25t dataset. The following performance measures were used in this study:

(4)

(5)

(6)

(7)

where *TP *is the number of true positives (binding residues with positive predictions); *TN *is the number of true negatives (non-binding residues with negative predictions); *FP *is the number of false positives (non-binding residues but predicted as binding sites); and *FN *is the number of false negatives (binding residues but predicted as non-binding sites). Since the datasets used in this study are imbalanced, the overall accuracy alone could be misleading. For instance, a classifier could achieve ~85% accuracy by simply predicting all the residues as negatives. Thus, both sensitivity and specificity are also computed from prediction results. Furthermore, the average of sensitivity and specificity, referred to as strength in this paper, may provide a fair measure of classifier performance as shown in previous studies [4,9].

The Receiver Operating Characteristic (ROC) curve is probably the most robust approach for classifier evaluation and comparison [15]. The ROC curve is drawn by plotting the true positive rate (*i.e.*, sensitivity) against the false positive rate, which equals to (1 - specificity). In this work, the ROC curve has been generated by using different threshold values for the output of a classifier and plotting the true positive rate against false positive rate for each threshold value. The area under the ROC curve (AUC) can be used as a reliable measure of classifier performance [16]. Since the ROC plot is a unit square, the maximum value of AUC is 1, which is achieved by a perfect classifier. Weak classifiers and random guessing have AUC values close to 0.5.

## Results and discussion

### Random forests for sequence-based prediction of DNA-binding residues

As the first step to develop the new approach for DNA-binding site prediction, random forests (RFs) were trained with three biochemical features that were used to construct ANN and SVM predictors in our previous studies [9,10]. The biochemical features, including the hydrophobicity index (feature *H*), side chain pK_a _value (*K*) and molecular mass (*M*) of an amino acid, were shown to provide relevant information for predicting DNA-binding residues [9]. The input vector contained 33 feature values because each data instance was a subsequence of eleven consecutive residues with the target residue in the middle position (see Methods). The context information provided by the ten neighbouring residues was found to be optimal for DNA-binding site prediction [9,10].

As shown in Table 1, the RF classifier constructed without evolutionary information achieved 70.23% overall accuracy with 73.46% sensitivity and 69.68% specificity in fivefold cross-validation experiments on the PDNA-62 dataset. Since the dataset was imbalanced with only 15% of the amino acid residues as DNA-binding sites, the performance of the RF classifier was also measured by the average of sensitivity and specificity (prediction strength = 71.57%), and the area under the receiver operating characteristic curve (ROC AUC = 0.7837). Different training parameters were tested for constructing the RF classifier, and the above performance measures were obtained with 1000 decision trees in the forest and *m *= 5 (see below).

**Table 1:** Performance of different classifiers constructed using biochemical features.

Classifier	Accuracy (%)	Sensitivity (%)	Specificity (%)	Strength (%)	ROC AUC
RF	70.23	73.46	69.68	71.57	0.7837
SVM	70.31	69.40	70.47	69.94	0.7524
ANN	64.38	71.33	63.51	67.42	0.7306

The results suggest that, with the three biochemical features, the RF classifier is slightly more accurate than the ANN and SVM predictors [9,10]. By using the same dataset (PDNA-62), the ANN and SVM predictors achieved the prediction strength of 67.42% and 69.94%, respectively. The ROC AUC of the ANN and SVM predictors is also less than that of the RF classifier. However, RFs have the major advantage in handling a large number of input variables should various descriptors of evolutionary information be used for input encoding.

### Improved classifier performance by using evolutionary information

Three types of evolutionary information, including the BLAST-based conservation score, position-specific scoring matrices (PSSMs), and the means and standard deviations of biochemical feature values, have been examined for their effect on classifier performance. The conservation score (*B*) was previously used to train ANNs for DNA-binding site prediction [9]. As shown in Table 2, the prediction strength and ROC AUC are slightly improved by adding the *B *score to the three biochemical features, suggesting that the conservation score does not capture all the evolutionary information for sequence-based prediction of DNA-binding residues.

**Table 2:** Effect of evolutionary information on the performance of RF classifiers.

Evolutionary information	Accuracy (%)	Sensitivity (%)	Specificity (%)	Strength (%)	ROC AUC
None	70.23	73.46	69.68	71.57	0.7837
PSSM	75.09	79.26	74.38	76.82	0.8521
*H*_*m*_, *H*_*d*_, *K*_*m*_, *K*_*d*_	74.78	77.70	74.29	75.99	0.8422
PSSM, *H*_*m*_, *H*_*d*_, *K*_*m*_, *K*_*d*_	78.20	78.06	78.22	78.14	0.8605

However, RF classifier performance is significantly improved by using the PSSM descriptor of evolutionary information. PSSMs were derived from the PSI-BLAST search against the UniProtKB database as described in Methods. Because each residue was encoded with 20 PSSM scores and 3 biochemical features, the input vector contained 253 values for a data instance with eleven residues. As shown in Table 2, the use of PSSM for input encoding improved the prediction strength to 76.82%. The classifier also had higher ROC AUC (0.8521) than the RF classifier constructed using the three biochemical features alone (AUC = 0.7837). The results were obtained with 1000 decision trees in the forest and the training parameter *m *set to 15. Other parameter settings were also tested, but did not give rise to better classifier performance.

The means (*H*_*m*_, *K*_*m *_and *M*_*m*_) and standard deviations (*H*_*d*_, *K*_*d *_and *M*_*d*_) of the three biochemical features represent new descriptors of evolutionary information, which indicate how well the biochemical properties of an amino acid position are conserved in the sequence alignment from the PSI-BLAST search. It was found that the use of *H*_*m*_, *K*_*m*_, *H*_*d*_, and *K*_*d *_in classifier construction improved the prediction strength to 75.99% with AUC = 0.8422 (Table 2). The RF classifier was constructed using 1000 decision trees and *m *= 8. However, adding *M*_*m *_and *M*_*d *_to the input vector did not result in further improvement of classifier performance (data not shown).

Interestingly, the most accurate classifier was obtained with a combination of PSSM, *H*_*m*_, *H*_*d*_, *K*_*m *_and *K*_*d *_in addition to the three biochemical features for input encoding. Since the input vector had 297 variables (27 inputs for each of the eleven residues in a data instance), the training parameter *m *was set to 17 for the forest with 1000 decision trees. As shown in Table 2, the resulting classifier achieved the overall accuracy of 78.20% with 78.06% sensitivity and 78.22% specificity. The prediction strength reached 78.14%, representing an increase of 6.57% when compared with the performance achieved without evolutionary information (71.57%). This RF also had the highest level of ROC AUC (0.8605) among all the classifiers (Table 2).

**Figure 2:**
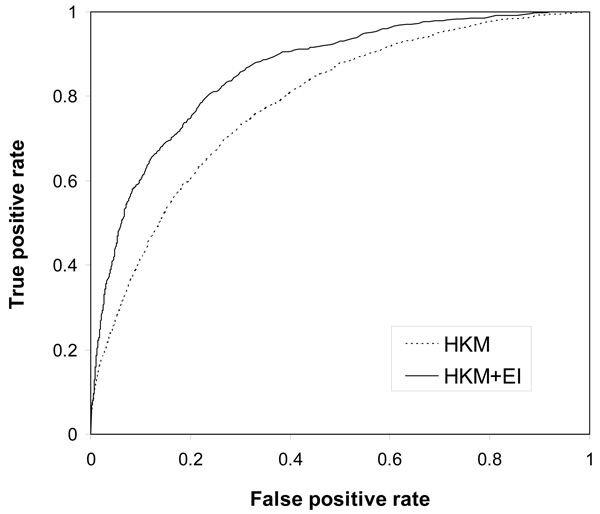
**ROC curves to show the effect of evolutionary information**. HKM represents the random forest classifier trained with the three biochemical features (*H*, *K *and *M*), and HKM+EI indicates the most accurate classifier using evolutionary information (PSSM, *H*_*m*_, *H*_*d*_, *K*_*m *_and *K*_*d*_).

The significant improvement of classifier performance by using evolutionary information has further been demonstrated in the ROC analysis (Figure 2). The ROC curves have been generated by varying the output threshold of RF classifiers, and each point on a ROC curve represents a trade-off between sensitivity and specificity. For classifier performance comparison, the ROC curve of a more accurate classifier is closer to the left-hand and top borders of the plot. As shown in Figure 2, the RF classifier trained with the two types of evolutionary information (HKM+EI) is clearly better than the classifier constructed using only biochemical features (HKM).

### Comparison of classifier performance using a separate test dataset

To further demonstrate the improved prediction of DNA-binding residues, the most accurate RF (also called BindN-RF) has been compared with the previous classifiers (BindN, DP-Bind and DBS-PSSM) using a separate test dataset, PDC25t. BindN uses the SVM classifier constructed using the three biochemical features (*H*, *K *and *M*) in our previous study [10]. The DP-Bind web server  provides PSSM-based SVM and kernel logistic regression predictors for DNA-binding site prediction [7,8]. DBS-PSSM  is the ANN predictor trained with PSSM and sequence information [6]. All the above classifiers have been constructed using the same training dataset, PDNA-62, which shares less than 25% sequence identity with the PDC25t dataset.

As shown in Table 3, BindN-RF gives the best predictive performance with the prediction strength at 76.86% and ROC AUC equal to 0.8495. Importantly, the performance measures achieved by BindN-RF on the separate test dataset (PDC25t) are comparable with those from the fivefold cross-validation (Table 2), suggesting that over-fitting has been avoided in the construction of the RF classifier. DP-Bind also gives relatively high performance with 73.32% prediction strength and ROC AUC = 0.8149 on the PDC25t dataset. However, the ANN predictor trained with PSSM and sequence information (DBS-PSSM) shows very low performance on the PDC25t dataset, probably owing to poor generalization of the representative DNA-binding residues in the relatively small training dataset (PDNA-62).

**Table 3:** Comparison of classifier performance using a separate test dataset.

Classifier	Accuracy (%)	Sensitivity (%)	Specificity (%)	Strength (%)	ROC AUC
BindN-RF	80.00	73.08	80.63	76.86	0.8495
BindN	70.81	68.70	71.01	69.85	0.7648
DP-Bind	78.89	65.89	80.76	73.32	0.8149
DBS-PSSM	67.91	37.48	70.72	54.10	0.5528

**Figure 3:**
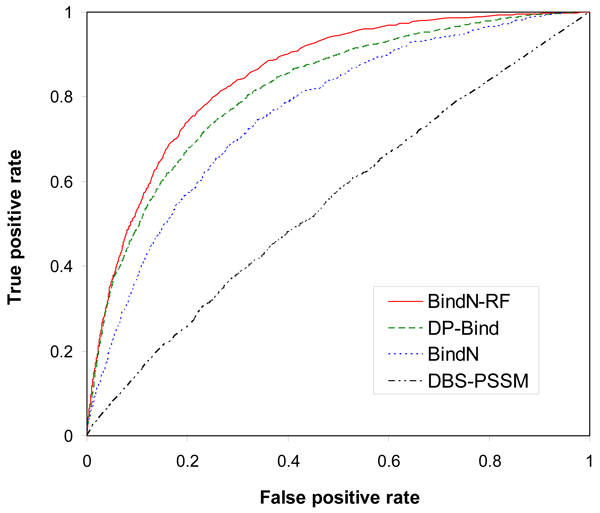
**ROC curves of different classifiers for DNA-binding site prediction**. The performance comparison is based on the PDC25t test dataset. The four different classifiers are BindN-RF (this study), BindN [10], DP-Bind [7,8] and DBS-PSSM [6].

In Figure 3, ROC curves have been generated for the four classifiers (BindN-RF, BindN, DP-Bind and DBS-PSSM) based on their predictions made for the PDC25t test dataset. Clearly, the RF classifier (BindN-RF) shows the best performance for almost all the trade-offs between sensitivity and specificity. The results suggest that the RF-based approach developed in this work is better than the previous methods for sequence-based prediction of DNA-binding residues.

### Structural validation of predicted DNA-binding residues

**Figure 4:**
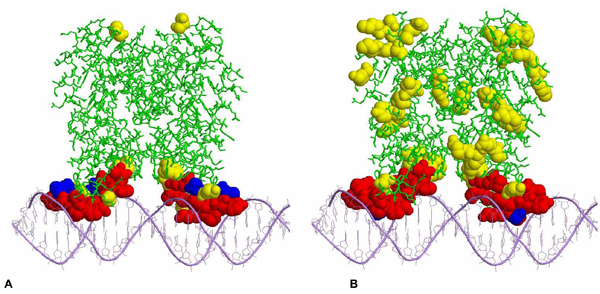
**Predicted DNA-binding residues shown in the context of three-dimensional structures**. Putative DNA-binding residues were predicted for the bacterial transcriptional regulator QacR (PDB ID: 1JT0) using BindN-RF (A) and BindN (B). In each protein-DNA complex, true positives (correctly predicted DNA-binding residues) are in red spacefill; true negatives in green wireframe; false positives in yellow spacefill; false negatives in blue spacefill; and the DNA double helix in purple.

To determine whether the prediction results can provide useful information for understanding protein-DNA interactions and that the RF-based approach is better than our previous method (BindN), the predicted DNA-binding residues have been examined in the context of three-dimensional structures. Figure 4 shows representative predictions made by BindN-RF and BindN for the 81 sequences in the PDC25t test dataset. The predictions were based solely on amino acid sequence information, and the structural data were used only for visualization of the prediction results. In Figure 4A, DNA-binding residues have been predicted using the RF classifier (BindN-RF) for the bacterial QacR protein (PDB ID: 1JT0) involved in multidrug binding and transcriptional regulation [17]. Significantly, 10 of the 12 DNA-binding residues were correctly predicted, and there were only 5 false positive predictions for the 166 non-binding residues in each protein subunit. When the QacR sequence was analyzed using BindN, the false positive rate was very high (58 false positive predictions for the 166 non-binding residues) although all the 12 DNA-binding residues were correctly identified (Figure 4B). Thus, for experimental studies such as site-directed mutagenesis, the prediction result from BindN does not provide as much useful information as that from BindN-RF.

### The BindN-RF web server

To make the accurate RF classifier available to the biological research community, we have developed the BindN-RF web server . Users can enter an amino acid sequence in FASTA format, and specify the desired level of sensitivity or specificity for DNA-binding site prediction. For a query sequence, the system performs a three-iteration PSI-BLAST search against the UniProtKB database to extract evolutionary information as described in Methods. The RF classifier constructed in this work is then used to predict DNA-binding residues in the query sequence. The user-defined level of sensitivity or specificity is used to determine the output threshold for the RF classifier according to its ROC curve. Thus, users can choose a specificity level higher than the default value (85%) to reduce the number of false positive predictions. The output report of BindN-RF has been designed to be self-explanatory, and is similar to that of BindN. A detailed description about the report format can be found in our previous paper [10].

## Conclusion

A random forest-based approach has been described in this paper for predicting DNA-binding residues in protein sequences. Since random forests can handle a large number of input variables and avoid model overfitting, accurate classifiers have been constructed by combining biochemical features with several descriptors of evolutionary information for input encoding. The new descriptors developed in the present work have been shown to enhance classifier performance when they are used together with the biochemical features and position-specific scoring matrices. Thus, the new descriptors capture certain evolutionary information that is not contained in position-specific scoring matrices previously used for DNA-binding site prediction. The best random forest classifier achieved 80.00% overall accuracy with 73.08% sensitivity and 80.63% specificity on a separate test dataset. Predictions at this level of accuracy may provide useful information for protein-DNA docking and experimental studies such as site-directed mutagenesis for understanding protein-DNA interactions. The new approach has been implemented in the BindN-RF web server for online prediction of DNA-binding residues in protein sequences.

## Competing interests

The authors declare that they have no competing interests.

## Authors' contributions

LW initiated the study, conducted the data analysis, and drafted the manuscript. MQY and JYY participated in result interpretation and manuscript preparation.
